# Effect of big five personality dimensions on the academic performance of college students

**DOI:** 10.3389/fpsyg.2025.1490427

**Published:** 2025-06-26

**Authors:** Rita Rani Bhattacharjee, Alajingi Ramkumar

**Affiliations:** Vellore Institute of Technology, Vellore, Tamilnadu, India

**Keywords:** personality, big five personality, students, academic performance, personality traits

## Abstract

**Introduction:**

Personality plays a crucial role in shaping the behavior of college students, impacting their academic performance, career prospects, and social relationships. This study examined the influence of the big five personality dimensions on students’ academic performance, specifically their grade point average (GPA).

**Methods:**

A purposive sampling method was used to analyze 384 students (220 males and 164 females) aged between 16 and 21 years from different family backgrounds (joint and nuclear families). Data were collected and analyzed using Statistical Package of Social Science (SPSS), categorizing students based on their GPA (<7 and >7) and assessing their personality traits.

**Results:**

Students with high conscientiousness demonstrated significantly better academic performance than those with high openness, agreeableness, extroversion, or neuroticism. While openness, extroversion, and agreeableness showed similar results, neuroticism harmed GPA.

**Discussion:**

These findings suggest that conscientiousness is a strong predictor of academic success, which is consistent with prior research on self-discipline and goal-setting. This study highlights the importance of personality traits in educational outcomes, which can be useful for academic counseling and student development programs.

## Introduction

Personality plays a vital role in the behavior of college students, and personality traits affect academic performance, placement, and sustainability in career and peer and family relationships. It is one of the major factors that determines a person’s lifestyle. Allport (1961) stated that personality is “the dynamic organization within the individual of those psycho-physical systems that determine his characteristic behavior and thought.” Personality refers to the consistency of who one is, has been, and will become. It also refers to the special blend of talents, values, hopes, love, hates, and habits that make each of us unique (Coon, 2006). Personality is a distinctive constant that cannot easily be changed. It influences emotions, thoughts, and even behaviors. The culture in which a child grows affects their personality (Sharma et al., 2024). Human personality is complex. Each individual possesses a unique blend of characteristics.

The culture in which a child grows affects the child’s personality. According to the American Psychological Association (2017), “Personality is defined as individual differences in characteristic patterns of thinking, feeling, and behaving.” Larsen and Buss (2018) state, “Personality is a stable, organized collection of psychological traits and mechanisms in the human being that influences his or her interactions with and modifications to the psychological, social and physical environment surrounding them.” Today, young people are caught in a never-ending race. Insistence on marks and grades has caused them to ignore their all-round personality development. Little or no time is spent on sports or personality development activities, such as yoga or meditation. The balance between the time spent on physical activity and education is not maintained. Today’s youth has become trapped in the digital world and electronic gadgets. Applications such as Facebook, Twitter (X), WhatsApp, YouTube, Instagram, and many online and offline games hold predominant places as youngsters are absorbed maximumly timewise. Students become more heavily invested in Internet access in nuclear families because their parents are busy with their office work and no other people are involved.

Kircaburun and Griffiths (2018) studied Instagram addiction and the Big Five personality traits: *The mediating role of self-liking*. They examined the relationships between personality, self-liking, daily Internet use, and Instagram addiction, as well as explored the mediating role of self-liking between personality and Instagram addiction using path analysis. In total, 752 university students completed a self-report survey that included the Instagram Addiction Scale (IAS), Big Five Inventory (BFI), and Self-Liking Scale. The results indicated that agreeableness, conscientiousness, and self-liking were negatively associated with Instagram addiction, whereas daily Internet use was positively associated with Instagram addiction.

Sugiarta and Dewi (2021) conducted a study on the correlation between the Big Five Personality traits and Internet addiction among 69 early adult individuals aged 18–32 years. The Internet Addiction Test (IAT) and Big Five Model—International Personality Item Pool—25 (BFM-IPIP-25) were used. Pearson’s correlation was used for data analysis to test their hypotheses. The findings showed a significant negative correlation between the Internet addiction premise dimensions of agreeableness and emotional stability. However, no correlation existed between the dimensions of extraversion, conscientiousness, and intellect. The results demonstrate that low emotional stability, such as in someone who exhibits irritable behavior or who experiences significant stress or mood chance, would be more susceptible to Internet addiction. Consequently, people with low agreeableness, such as someone who behaves evilly, cruelly, or harms others have a high Internet addiction value. Recent research has extensively examined the relationship between personality traits and academic performance.

However, few studies have explored this relationship within specific sociocultural frameworks. This study aimed to address this gap by investigating how family structure (nuclear vs. joint families) moderates the relationship between the Big Five personality traits and academic achievement among engineering students. By considering cultural and family backgrounds, this study offers practical insights that are applicable to academic counseling and student development programs.

### Big five personality test

The Big Five personality tests were used to assess students. Exploratory research in this field is currently underway. The Big Five personality test has five wide-range areas that describe human personas and justify individual variances. Many scholars and researchers have explored and elucidated five domains based on experimental and data-driven research. Tupes and Christal (1992) advanced the initial model based on the work conducted at the U. S. Air Force Personnel Laboratory in the late 1950s. Digman (1990) proposed his five-factor personality model in 1990 and Goldberg (1993) extended it to the highest level of organizations in 1993. Five traits (OCEAN) in the test were recognized as the basic personality dimensions that determine a person’s behavior. The Big Five personality traits are openness, conscientiousness, extraversion, agreeableness, and neuroticism” (OCEAN; Van Thiel, 2018). Since the personality of students is more affected by previously discussed factors, the present research was conducted to explore the effects of the Big Five Personality Dimensions on students’ academic performance in the present scenario.

## Literature reviews

Wang et al. (2023) verified the influence of personality traits on students’ academic achievement and assessed the mediating effects of major identity and self-efficacy under the classical model of chain-mediating effects using data from business major students. The results show that both extraversion and conscientiousness have a positive overall effect on students’ academic achievement, primarily through the chain-mediating effects of and major identity and self-efficacy. This mediation is mainly attributed to the impact of self-efficacy, which is more obvious in the dimension of behavioral efficacy. The overall effect of an agreeable personality on academic achievement is negative and is mainly reflected through a direct effect. John et al. (2020) investigated the impact of the Big Five personality traits on the academic performance of university students in terms of their CGPA using the 20-item short Mini-IPIP Five-Factor personality test developed by Donnellan et al. (2006). They conducted a study on 406 undergraduate students at the Forman Christian College, Lahore. The results confirm the predictive validity of the Big Five personality traits. Openness was most positively related to academic performance, followed by agreeableness and conscientiousness. However, neuroticism and extraversion were not significantly correlated with academic performance.

Bhagat et al. (2019) studied the relationship between the Big Five Personality Traits and Academic Performance in Medical Students. They conducted a cross-sectional study on 122 first- and second-year medical students at the University of Sultan, Zainal Abidin, Malaysia. All the Big Five Personality traits positively predicted students’ overall grades. Of all the personality traits, openness and neuroticism were most positively related to students’ academic achievement and predicted their overall grades. The students’ traits of agreeableness and conscientiousness showed no significance. However, extraversion was positively related but not statistically significant. Personality traits, particularly conscientiousness, have been consistently linked to academic success (Mammadov, 2022).

However, gender differences may moderate these relationships because gender roles in different cultures shape personality development and academic engagement. In the current sample, males (57.3%) and females (42.7%) were almost equally represented, prompting the consideration of gender in future studies. Family structure also plays a significant role in personality development. In this study, 72.9% of participants were from nuclear families, whereas 27.1% were from joint families. The term “nuclear family” refers to a family unit consisting of parents and their immediate children, while “joint family” includes extended relatives living together. Previous literature has sparsely discussed how these family types impact student outcomes, representing a significant gap that this study seeks to address.

The study concluded that the Big Five Personality Traits were associated with the Academic Performance of Medical Students. Tomsik (2018) conducted research on the outcomes of the Big Five personality behaviors. He studied the educational performances of college students and found that, with an increase in age, individuals’ personality traits and Partial Least Squares were used to find the essential associations concerning the collected figures had a smaller effect on educational performance. In his proposed research, university students of I grade (*N* = 402) completed the Five-Factor Inventory and informed them of their grade point average (GPA). Furthermore, conscientiousness positively influenced students’ academic performance. A meta-analysis by Mammadov (2022) offered a critical, large-scale synthesis of the relationship between the Big Five personality traits and academic performance at all educational levels. It combines the results of 267 separate groups of participants (samples) from 228 different research studies, with a total of 413,074 participants. It established conscientiousness as the strongest and most consistent predictor of academic success, surpassing previous meta-analyses in terms of size and reliability. The study also revealed the nuanced moderating effects of educational level and cultural context, providing a deeper understanding of how traits such as openness and agreeableness vary in their influence over time. Its robust findings underscore the importance of conscientiousness not just as a distal trait but as a pivotal factor linked to academic motivation, effort, and achievement across diverse educational settings. Rajapakshe (2017) conducted a study to define the outcome of Big Five personality behaviors on Sri Lankan pupils’ educational accomplishments in a private higher educational institute in Sri Lanka.

In total, 200 participants were selected. Two equal groups were formed to conduct the Discriminant Analysis; GPA below 2.00 and above 2.00 and trial size decreased to 116. Based on the outcomes of this study, neuroticism is an important predictor of academic attainment among the other five traits. Moreover, the study reveals that conscientiousness has subsequently become a variable of extraversion. Therefore, conscientiousness has a constructive affiliation with the two groups of students’ educational scores, whereas each of the other four personality traits has been identified to be negatively related to the educational performance of both student groups. Papamitsiou and Economides (2014) conducted research on BFI using temporal learning analytics. They studied the effect of personality traits on student performance during computer-based testing and investigated the effects of conscientiousness and extraversion on student performance. Ninety-six respondents completed the Big Five questionnaire to record their personality traits. Partial Least Squares analysis was used to determine the essential associations between the collected figures. The major outcomes showed a productive effect of conscientiousness on certainty and a constructive outcome of extraversion on the expectancy of goals. The relationship between family type (nuclear vs. joint) and personality traits using the BFI revealed that joint family students scored higher on extraversion and agreeableness, whereas nuclear family students showed greater openness and neuroticism. Conscientiousness remained similar in both the groups (Sahu, 2022).

### Objectives

This study aimed to study the effects of the Big Five personality dimensions on the age, gender, GPA, and academic performance of college students.

### Hypotheses

A significant difference exists between the Big Five personality dimensions based on age, gender, GPA, and academic performance of college students.

## Materials and methods

**Table:** 

No. of participants	Gender	Participants	Age	Participants
384	Male	220	16–18 years	260
	Female	164	19–21 years	124

### Instruments

The Big Five Personality Inventory was developed by Goldberg (1993). The inventory contains 44 questions to measure the Big Five personality dimensions: openness, agreeableness, conscientiousness, extroversion, and neuroticism. The questionnaire used the following five rating scales: disagree, slightly disagree, neutral, slightly agree, and agree. It has reliability values ranging from 0.70 and 0.90 and its validity is above 0.80.

### Academic performance

Participants’ grades were obtained along with the questionnaire, with an option for demographic details. The semester-basis grade system was obtained as per their batch was collected individually with their consent.

### Process and participants

This study used a purposive sampling method on 600 first-year students, where 384 students were randomly selected based on their interest in and consent to a personality test from private college in the Vellore District, Tamil Nadu. The selected students received explanations regarding the questionnaire (the Big Five Personality Inventory) as well as the five dimensions of personality that would be measured for all interested students. The students were informed that there were no right or wrong answers, and they were asked to respond immediately after reading the statements. Informed consent was obtained from each participant prior to the study. The qualities of each personality dimension were explained and participants were asked to complete the questionnaire. They were very interested in measuring their own personalities, and their doubts were clarified whenever required. Students scored differently on the five dimensions of the personality inventory, and their interpretations were recorded in the results.

### Statistical analysis

The Statistical Package of Social Science (SPSS) (IBM 20) was used to enter and analyze the data collected from the students personally. Percentage analysis was performed using age, gender, and type of family as demographic variables. The mean and standard deviation were calculated based on the GPA, gender, and family status of the selected students based on the five personality dimensions. Finally, a correlation coefficient was calculated for the five personality dimensions to measure the significance level of each dimension separately.

### Ethics

This study involved humans as the subjects. We confirmed that all procedures performed in the studies involving human participants were based on the ethical standards of the institutional research committee and the 1964 Helsinki Declaration and its later amendments. Informed consent was obtained from all students involved in the study.

## Results

**Figure 1:**
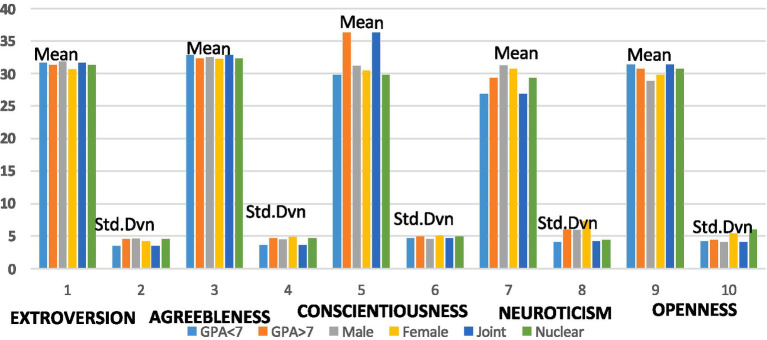
Comprehensive graphical report of Big 5 personality traits data.

The comparative statistical analysis of this study is presented in Figure 1, along with gender, age, and GPA-based response means and standard deviations.

Table 1 shows the demographic details of college students in Vellore District. They were categorized according to factors such as age, gender, and family type using the Big Five personality trait questionnaire. Among the 384 volunteers, 67.7% were between the ages of 16 and 18 and 32.3% were between the ages of 19 and 21. In terms of gender, 57.3% were male students and 42.7% were female students. In terms of family type, 27.1% belonged to joint families and 72.9% were from nuclear families. The results show a Measurement Reliability and Internal consistency for the Big Five dimensions with extroversion (*α* = 0.78), agreeableness (α = 0.81), conscientiousness (α = 0.84), neuroticism (α = 0.79), and openness (α = 0.76). The Inferential Statistics with Independent samples t-tests showed that students with GPA > 7 scored significantly higher on conscientiousness than students with GPA < 7 (*p* < 0.001). Students from joint families scored higher in conscientiousness and displayed lower neuroticism than did students from nuclear families (*p* < 0.05).

**Table 1:** Demographic details of college students on big five personality dimensions.

Age			Gender			Type of family		
	Frequency	Percent		Frequency	Percent		Frequency	Percent
16–18	260	67.7	Male	220	57.3	Joint	104	27.1
19–21	124	32.3	Female	164	42.7	Nuclear	280	72.9
Total	384	100	Total	384	100	Total	384	100

As shown in Table 2, the GPA (below 7 and above 7) of the students was considered for measuring the Big Five Personality Inventory and determining the mean and standard deviation of various dimensions. Students, both below 7 and above 7 seemed to have similar means, except for the conscientious dimension of the personality factor. The students scoring below 7 GPA were not methodical in their study; therefore, they had low scores, because they were emotionally unstable with negative emotions about their future, but were more open and friendly than the students who scored above 7 GPA. Komarraju et al. (2009) studied the role of the Big Five personality traits in predicting college students’ academic motivation and achievement; 308 undergraduate college students completed the Five-Factor Inventory and Academic Motivations Scale and reported their college grade point average (GPA). A correlation analysis revealed significant relationships. Additionally, regression analyses indicated that conscientiousness and openness explained 17% of the variance in intrinsic motivation, conscientiousness and extraversion explained 13% of the variance in extrinsic motivation, and conscientiousness and agreeableness explained 11% of the variance in a motivation. Four personality traits (conscientiousness, openness, neuroticism, and agreeableness) explained 14% of the variance in GPA, and intrinsic motivation to accomplish things explained 5% of the variance. Finally, conscientiousness emerged as a partial mediator in the relationship between intrinsic motivation and GPA.

**Table 2:** Mean and standard deviation of big five personality dimensions in terms of GPA among college students.

GPA	Extroversion		Agreeableness		Conscientiousness		Neuroticism		Openness	
	*M*	SD	*M*	SD	*M*	SD	*M*	SD	*M*	SD
Less than 7	31.63	3.49	32.85	3.58	29.75	4.69	26.81	4.05	31.37	4.18
More than 7	31.35	4.47	32.29	4.67	36.26	4.95	29.30	6.03	30.65	4.39

In Table 3, the means of all personality traits of the Big Five Personality Dimensions are shown to be similar in terms of gender, whereas females seem to be more open to imagination and learning new things than are males. In terms of gender, the students are more familiar with the traits of this personality inventory.

**Table 3:** Mean and standard deviation of big five personality dimensions in terms of gender among college students.

Gender	Extroversion		Agreeableness		Conscientiousness		Neuroticism		Openness	
	*M*	SD	*M*	SD	*M*	SD	*M*	SD	*M*	SD
Male	31.85	4.60	32.49	4.46	31.20	4.531	31.29	5.94	28.87	4.02
Female	30.59	4.19	32.22	4.88	30.44	5.089	30.71	7.28	29.76	5.42

In Table 4, the personality traits of extroversion, agreeableness, and openness are almost similar among the students belonging to joint and nuclear families with means of 31.63 and, 31.35, 32.85 and 32.29, and 31.37 and 30.65, respectively, conveying similarity in all the three personality traits in terms of family type. Consequently, it is extreme in the case of personality traits of conscientiousness in students belonging to joint and nuclear families with means of 36.26 and 29.75, respectively, whereas the personality traits of neuroticism show some differences among the students in terms of joint and nuclear families with means of 26.81, and 29.30, respectively. Students belonging to joint families seem to be more organized because of the guidance of their grandparents, whereas those belonging to nuclear families are single children with temper tantrums. This is because of the collective responsibility and social harmony in the joint family system.

**Table 4:** Mean and standard deviation of big five personality dimensions in type of family among college students.

Type of family	Extroversion		Agreeableness		Conscientiousness		Neuroticism		Openness	
	*M*	SD	*M*	SD	*M*	SD	*M*	SD	*M*	SD
Joint	31.63	3.49	32.85	3.58	36.26	4.69	26.81	4.18	31.37	4.05
Nuclear	31.35	4.47	32.29	4.67	29.75	4.95	29.30	4.39	30.65	6.03

In Table 5, the results show a positive relationship among the personality traits at either the 0.05 or 0.01 level of Pearson correlation, except for conscientiousness, agreeableness, openness, and neuroticism. The family plays a major role in building an individual’s personality. Based on the type of family, it is understood that students who come under the personality dimension of conscientiousness easily believe in people. They are also more emotional, sympathetic, and creative.

**Table 5:** Correlations between personality dimensions among college students.

Correlations		Extroversion	Agreeableness	Neuroticism	Conscientious	Openness
Extroversion	Pearson correlation	1	0.316^**^	0.384^**^	0.237^*^	0.345^**^
	Sig. (two-tailed)		0.002	0.000	0.020	0.001
	N	384	384	384	384	384
Agreeableness	Pearson correlation	0.316^**^	1	0.354^**^	0.065	0.289^**^
	Sig. (two-tailed)	0.002		0.000	0.528	0.004
	*N*	384	384	384	384	384
Neuroticism	Pearson correlation	0.384^**^	0.354^**^	1	0.194	0.369^**^
	Sig. (two-tailed)	0.000	0.000		0.058	0.000
	N	384	384	384	384	384
Conscientiousness	Pearson correlation	0.237^*^	0.065	0.194	1	0.134
	Sig. (two-tailed)	0.020	0.528	0.058		0.193
	*N*	384	384	384	384	384
Openness	Pearson correlation	0.345^**^	0.289^**^	0.369^**^	0.134	1
	Sig. (two-tailed)	0.001	0.004	0.000	0.193	
	*N*	384	384	384	384	384

**Correlation is significant at the 0.01 level (two-tailed). *Correlation is significant at the 0.05 level (two-tailed).

## Discussion

Students with a GPA higher than 7 demonstrated greater conscientiousness (*M* = 36.26, SD = 4.95) than those with a GPA lower than 7 (*M* = 29.75, SD = 4.69). Extraversion, agreeableness, and openness were moderately positively correlated, suggesting that these traits co-occur among students. Moreover, students from joint families exhibited higher mean scores for conscientiousness and lower scores for neuroticism than those from nuclear families, indicating that family environment may influence personality development in ways that impact academic success. As shown in Table 5, the correlation matrix revealed significant relationships among the Big Five personality traits—extraversion, agreeableness, neuroticism, conscientiousness, and openness—among 384 participants. Extraversion shows moderate positive correlated with agreeableness (*r* = 0.316, *p* = 0.002), neuroticism (*r* = 0.384, *p* = 0.000), conscientiousness (*r* = 0.237, *p* = 0.020), and openness (*r* = 0.345, *p* = 0.001), indicating that extroverted individuals tended to be more agreeable, emotionally reactive, conscientious, and open to new experiences. Agreeableness also positively correlated with neuroticism (*r* = 0.354, *p* = 0.000) and openness (*r* = 0.289, *p* = 0.004), which is an interesting finding because agreeableness is typically linked to emotional stability rather than neuroticism. Neuroticism shows a moderate correlation with openness (*r* = 0.369, *p* = 0.000), suggesting that individuals with high neuroticism may be more open to experiences.

However, conscientiousness was not significantly correlated with agreeableness (*r* = 0.065, *p* = 0.528) or neuroticism (*r* = 0.194, *p* = 0.058), indicating that it may be influenced by other factors. Similarly, openness was strongly correlated with extraversion, agreeableness, and neuroticism. However, its relationship with conscientiousness (*r* = 0.134, *p* = 0.193) was weak and statistically insignificant. Overall, the findings suggest that extraversion, neuroticism, and openness are the most interconnected traits, whereas conscientiousness remains relatively independent. Some unexpected results, such as the positive correlation between agreeableness and neuroticism, require further investigation to understand the underlying influences. These findings align with previous literature, confirming conscientiousness as a robust predictor of academic success (Mammadov, 2022). In addition, the influence of family structure appears significant; students from joint families demonstrated personality profiles that were more conducive to academic success, possibly because of stronger social support systems and shared responsibilities typical of extended family living. Gender differences, which are not the focus of the current analysis, are acknowledged as potential moderating factors, suggesting avenues for future research. This study also emphasizes the importance of considering sociocultural factors when developing educational interventions and counseling strategies.

### Limitations

The present study used a purposive sampling method with a limited number of students therefore, the study cannot be generalized. The importance of other methods, such as experimental and clinical trials, has not been highlighted, and this needs to be considered in future studies. Practical implications include the use of personality assessments for academic advice and the development of intervention programs tailored to students’ family backgrounds. Although the sample was limited to college students, which restricts generalizability, the findings provide a strong basis for future research.

### Scope for further research

More respondents can be included in further research so that future generations will gain a better understanding of their personality traits, which may help them lead a better life. Longitudinal studies examining the dynamic development of personality traits across educational disciplines and family structures are recommended. Future research should also explicitly analyze gender moderation effects and cultural influences on the personality–academic performance link.

## Conclusion

The traits of the Big Five Personality Dimensions helped analyze college student behaviors in terms of age, gender, family type, and academic performance. Some psychological issues or personality disorders are due to family disturbances, single parents, a lack of understanding among the members of the family, or forcing the students to perform as per parental guidelines. This study reinforces the critical role of personality traits, particularly conscientiousness, in the academic achievement of engineering students. Furthermore, it highlights how the family structure can subtly shape personality traits linked to academic outcomes. The present study mainly focused on students’ relationships with their family members, peers, relatives, seniors, teachers, and society, among others, using the BFI (conscientiousness, openness, extroversion, neuroticism, and agreeableness). In this study, the mean of conscientiousness differed in terms of GPA and family type (joint and nuclear). The students in the category of lower GPA are not as organized when compared to the students with higher GPA; similarly, the students who come from a joint family appear to be more organized, as juxtaposed with students belonging to the nuclear family. Students from nuclear families may feel deprived of love and affection from their parents as both are occupied with their careers. Being in nuclear families, they also lack frequent contact with their grandparents. Establishing an amicable relationship and spending quality time listening to and talking to them will help parents understand their children. This will refine their behavior, create self-awareness, help them utilize their potential, and address their weaknesses. All these collectively assure calm and peace in the family and relationships and promote a better way of living.

## Data Availability

The raw data supporting the conclusions of this article will be made available by the authors, without undue reservation.

## References

[ref9001] Allport G. W. (1961). Pattern and growth in personality. New York: Holt, Rinehart & Winston.

[ref1] American Psychological Association. (2017). Personality. Available online at: https://www.apa.org/topics/personality. (Accessed 4 September 2022)

[ref2] Bhagat V. Shetty C. K. Husain R. Mat K. C. Simbak N. B. Aung M. M. T.. (2019). The relationship between big five personality traits and academic performance in medical students. Res. J. Pharm. Technol. 12, 4189–4196. doi: 10.5958/0974-360X.2019.00721.2

[ref3] Coon D. (2006). Psychology: A modular approach to mind and behavior. 10th Eth Edn. Belmont, CA: Thomson Wadsworth.

[ref4] Digman J. M. (1990). Personality structure: emergence of the five-factor model. Annu. Rev. Psychol. 41, 417–440. doi: 10.1146/annurev.ps.41.020190.002221

[ref9002] Donnellan M. B. Oswald F. L. Baird B. M. Lucas R. E. (2006). The Mini-IPIP scales: Tiny-yet-effective measures of the Big Five factors of personality. Psychol. Assess. 18, 192–203. 16768595 10.1037/1040-3590.18.2.192

[ref5] Goldberg L. R. (1993). The structure of phenotypic personality traits. Am. Psychol. 48, 26–34. doi: 10.1037//0003-066x.48.1.26, PMID: 8427480

[ref6] John R. John R. Rao Z. R. (2020). The big five personality traits and academic performance. J. Law Soc. Stud. 2, 10–19. doi: 10.52279/jlss.02.01.1019

[ref7] Kircaburun K. Griffiths M. D. (2018). Instagram addiction and the big five of personality: the mediating role of self-liking. J. Behav. Addict. 7, 158–170. doi: 10.1556/2006.7.2018.15, PMID: 29461086 PMC6035031

[ref8] Komarraju M. Karau S. J. Schmeck R. R. (2009). Role of the big five personality traits in predicting college students’ academic motivation and achievement. Learn. Individ. Differ. 19, 47–52. doi: 10.1016/j.lindif.2008.07.001

[ref9] Larsen R. R. Buss D. M. (2018). Personality psychology: Domains of knowledge about human nature. New York: McGraw-Hill Education.

[ref10] Mammadov S. (2022). Big five personality traits and academic performance: a meta-analysis. J. Pers. 90, 222–255. doi: 10.1111/jopy.12663, PMID: 34265097

[ref11] Papamitsiou Z. Economides A.A. (2014). “The effect of personality traits on students’ performance during computer-based testing: a study of the big five inventory with temporal learning analytics,” in Proceedings – IEEE 14th international conference on advanced learning technologies, ICALT 2014

[ref12] Rajapakshe W. (2017). A study on the big five personality dimensions’ effect on university students’ academic performance. IOSR JBM. 19, 69–75. doi: 10.9790/487X-1912016975

[ref13] Sahu D. P. (2022). A comparative study of joint-nuclear family school children of different birth-order on extraversion. Int. J. Indian Psychol. 7, 417–440. doi: 10.25215/0703.033

[ref14] Sharma G. Naidoo J. M. Ihalanayake R. Joshi M. Tharapos M. (2024). Does culture influence the development of personality traits of accounting students at Indian universities? Acc. Educ. 1-28, 1–28. doi: 10.1080/09639284.2024.2429703, PMID: 40406382

[ref16] Sugiarta R. Dewi F. I. R. (2021). “The correlation between the big-five personality and internet addiction among early-adult individuals”, In Proceedings of the international conference on economics, business, social, and humanities (ICEBSH 2021), advances in social science, education and humanities research, 570

[ref17] Tomsik R. (2018). Impact of big five personality traits on academic performance of university students, in PHD existence 2018 ‘infinity in psychology’ conference (February 2018), at Olomouc, Czech Republic, 8.

[ref18] Tupes E. C. Christal R. E. (1992). Recurrent personality factors based on trait ratings. J. Pers. 60, 225–251. doi: 10.1111/j.1467-6494.1992.tb00973.x, PMID: 1635043

[ref19] Van Thiel E. (2018) Big 5 personality test traits. Available online at: https://www.123test.com/big-five-personality-theory/ (Accessed November 20, 2022).

[ref20] Wang H. Liu Y. Wang Z. Wang T. (2023). The influences of the big five personality traits on academic achievements: chain mediating effect based on major identity and self-efficacy. Front. Psychol. 14:1065554. doi: 10.3389/fpsyg.2023.1065554, PMID: 36777199 PMC9911834

